# Impact of Acetylated and Non-Acetylated Fucose Analogues on IgG Glycosylation

**DOI:** 10.3390/antib8010009

**Published:** 2019-01-10

**Authors:** Martina Zimmermann, Janike Ehret, Harald Kolmar, Aline Zimmer

**Affiliations:** 1 Merck Life Sciences, Upstream R&D, Frankfurter Strasse 250, 64293 Darmstadt, Germany; Janike.ehret@merckgroup.com (J.E.); Aline.zimmer@merckgroup.com (A.Z.); 2 Institute for Organic Chemistry and Biochemistry, Technische Universität Darmstadt, Alarich-Weiss-Strasse 4, 64287 Darmstadt, Germany; Kolmar@Biochemie-TUD.de

**Keywords:** acetylation, CHO cell culture, fucose analogues, IgG fucosylation, incorporation

## Abstract

The biological activity of therapeutic antibodies is highly influenced by their glycosylation profile. A valuable method for increasing the cytotoxic efficacy of antibodies, which are used, for example, in cancer treatment, is the reduction of core fucosylation, as this enhances the elimination of target cells through antibody-dependent cell-mediated cytotoxicity. Development of fucose analogues is currently the most promising strategy to reduce core fucosylation without cell line engineering. Since peracetylated sugars display enhanced cell permeability over the highly polar free hydroxy sugars, this work sought to compare the efficacy of peracetylated sugars to their unprotected forms. Two potent fucose analogues, 2-deoxy-2-fluorofucose and 5-alkynylfucose, and their acetylated forms were compared for their effects on fucosylation. 5-alkynylfucose proved to be more potent than 2-deoxy-2-fluorofucose at reducing core fucosylation but was associated with a significant higher incorporation of the alkynylated fucose analogue. Acetylation of the sugar yielded only slightly lower fucosylation levels suggesting that acetylation has a minor impact on cellular entry. Even though the efficacy of all tested components was confirmed, results presented in this study also show a significant incorporation of unnatural fucose analogues into the glycosylation pattern of the produced IgG, with unknown effect on safety and potency of the monoclonal antibody.

## 1. Introduction

Recombinant monoclonal antibodies (mAbs) are commonly produced using suspension cultures of Chinese hamster ovary (CHO) cells. The mammalian CHO cell line can produce bioactive therapeutics, which are mostly non-immunogenic in humans [1,2]. Along with aggregate formation, low molecular weight species, charge variants and misincorporation of amino acids in the protein backbone, the glycosylation of recombinant produced antibodies is a main critical quality attribute (cQA).

Changes to the glycosylation profile of mAbs can have a strong impact on various aspects of their biological activity as summarized in several reviews [3,4,5]. This is particularly relevant for therapeutic antibodies engineered for cancer treatment, for which the mechanism of action implicates binding to the target cancer cell and endogenous natural killer (NK) cells that are responsible for the effector function through antibody-dependent cell-mediated cytotoxicity (ADCC). Therapeutic antibodies lacking core fucose in the Fc-linked glycan display enhanced ADCC activity compared to highly-fucosylated variants [3], through significant higher binding affinity of afucosylated IgG to the respective FcγRIIIα of NK cells [6,7,8]. Several fucose deficient antibodies are currently in clinical studies as reported recently [9].

The enzyme fucosyltransferase 8 (FUT8) catalyzes the transfer of fucose residues onto the glycan core structure from guanosine diphosphate fucose (GDP-fucose). Protein fucosylation was shown to be reduced by addition of fluorinated fucose analogues, via their actions as inhibitors of FUTs [10]. In cell-free assays, initial experiments showed family-specific inhibition of FUT with GDP-2-deoxy-2-fluorofucose (GDP-2F-Fuc) and GDP-6-fluorofucose (GDP-6F-Fuc) [11]. Rillahan et al. later reported the specific reduction of FUT8-mediated core fucosylation through addition of fucose analogues to cell culture [10]. They demonstrated that the fluorinated fucose analogues are cell permeable and are converted via the salvage pathway into the corresponding donor substrates (GDP-2F-Fuc and GDP-6F-Fuc), which can compete with the actual FUT substrate GDP-Fuc. Intracellular accumulation of the fluorinated nucleotide sugars was shown to inhibit the de novo synthesis of GDP-Fuc by acting as a feedback inhibitor [10]. Peracetylated analogues were developed to further improve cell permeability and were shown to be deacetylated in the cell cytoplasm through the action of non-specific esterases [10,12]. Altogether, 2F-Peracetyl-fucose (2F-PerAcFuc) acts as a potent inhibitor due to the feedback inhibition and the high K_M_ value of GDP-2F-Fuc to FUT8 [10]. 5-alkynylfucose (5-AlkFuc) is another fucose analogue that has been shown to mediate a strong inhibition of FUT8 (>80%) [13,14]. Kizuka et al. proposed a mechanism of action involving the inhibition of GDP-4-keto-6-deoxymannose 3,5-epimerase-4-reductase (FX), an enzyme required for de novo synthesis of GDP-Fuc from GDP-mannose [15]. Incorporation of 5-AlkFuc in the glycan structure of the produced recombinant protein was observed by Hsu et al. [16].

**Figure 1:**
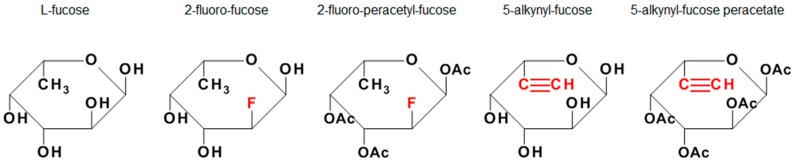
L-Fucose and its monosaccharide analogues used in this study. The key structural modifications are highlighted in red.

In this study, we performed a side-by-side comparison of the usage of 2F-Fuc, 5-AlkFuc and their acetylated forms (Figure 1) in fed-batch experiments using CHO cells producing an IgG1. The efficacy of the compounds in reducing the fucosylation was evaluated based on common cell culture data like viable cell density (VCD) and specific productivity as well as the glycosylation profile, including an estimation of the incorporation of the modified sugars into the oligosaccharides of the recombinant proteins.

## 2. Materials and Methods 

### 2.1. Reagents and Cell Line

5-alkynylfucose was purchased from Carbosynth (Compton, UK), 5-alkynylfucose peracetate from Thermo Fisher scientific (Waltham, MA, USA), 2-deoxy-2-fluorofucose from Cayman Chemical (Ann Arbor, MI, USA) and Dimethyl sulfoxide (DMSO) as well as 2F-peracetyl-fucose were purchased from Merck (Darmstadt, Germany). A CHOK1 cell line producing a recombinant IgG1 was used in this study.

### 2.2. Cell Culture Media and Process Conditions

Cells were cultivated in Cellvento^®^ 4CHO (Merck), a chemically defined CHO cell culture medium that contains all essential components for optimal cell growth and protein production. Cellvento^®^ 4Feed (Merck), a highly concentrated and neutral pH feed was used as a feeding medium, providing essential components for extended culture duration and productivity.

The fed-batch process was performed in 50 mL spin tubes with vented cap at 37 °C, 5% CO_2_, 80% humidity and a rotation speed of 320 rpm. 3% (*v*/*v*) of the starting volume was added on day three and 6% (*v*/*v*) of the total volume was added on day 5, 7 and 10. The glucose level was maintained by adding a specific amount of a 400 g/L glucose stock solution on demand to up to 6 g/L during the week and up to 12 g/L over the weekend. The non-supplemented feed served as a control condition and was compared to conditions containing the fucose analogues in the feed, and a DMSO-only condition. Concentrated stock solutions of the fucose analogues were prepared in DMSO due to reported water insolubility. The stock solution with 34.2 mM PerAcFuc was prepared according to the maximal solubility reported by the manufacturer leading to a total amount of 2.33% (*v*/*v*) DMSO in the feed. Stock solutions of 150 mM 2F-Fuc, 50 mM 5-AlkFuc and 50 mM 5-AlkFucPerAc were prepared similarly in DMSO. All inhibitor conditions and the DMSO control condition were adjusted to have a uniform DMSO concentration of 2.33% in the feed. Following inhibitor supplementation, the pH of all feeds was adjusted to neutral (pH 7.0 ± 0.2). Osmolality was measured by a cryoscopic osmometer (Gonotec, Berlin, Germany) and both cell culture media and feeds were sterile-filtered (0.22 µm) and stored at 4 °C, protected from light until usage.

Cell counts and viability were measured with a Vi-CELL™ XR 2.04 cell counter (Beckman Coulter, Fullerton, CA, USA). The inoculation density was 3.5 × 10^5^ cells/mL in 30 mL starting volume. Experimental conditions were performed with six replicates, whereby four replicates were used for spent media analysis and two additional replicates were used for glycosylation analysis. Spent media analysis including glucose, IgG, lactate dehydrogenase, ammonia, iron, lactate, phosphate and pyruvate was performed with the bioprocess analyzer CEDEX Bio HT (Roche, Mannheim, Germany) after centrifugation of the sample for 5 min at 4500 rpm (2287× *g*).

### 2.3. Antibody Purification and Analysis of the Glycosylation Pattern

The antibody was purified from the cell culture supernatant by protein A PhyTips^®^ with the semi-automated system Pure Speed (PhyNexus Inc., San Jose, CA, USA). The relative quantitative analysis of the N-linked glycans was performed after glycan cleavage from the antibody and derivatization using the GlycoWorks™ RapiFluor-MS™ N-Glycan Kit (Waters, Milford, MA, USA) and with ultra-performance liquid chromatography coupled to a mass spectrometer (UPLC-MS). UPLC analysis was performed according to the manufacturer protocol using an ACQUITY UPLC Glycan BEH Amide Column (300 Å, 1.7 µm, 2.1 × 150 mm) coupled to an ACQUITY UPLC^®^ FLR Detector (Ex: 265 nm, Em: 425 nm) from Waters. Identification of the glycan structures was performed using glycan standards to detect the specific retention time and MS spectra by calculating the mass to charge ratio with an electrospray ionization source in positive mode (Synapt G1 HDMS, Waters). A sample volume of 18 µL was injected into the system using a column temperature of 45 °C. Separation of the glycans was achieved using 50 mM ammonium formate (pH 4.4) and acetonitrile as solvents. The ratio of 50 mM ammonium formate to acetonitrile was increased during the UPLC from an initial 20:80 ratio (0 min) to 27:73 (3 min) and 37:63 (35 min) at a flowrate of 0.5 mL/min. 100% of 50 mM ammonium formate was reached between 36.5 min and 39.5 min at a reduced flowrate of 0.2 mL/min. A similar flowrate was used to shift again to the initial 20:80 ratio (43.1 min) and in the end, the flowrate was increased again to 0.5 mL/min using the same solvent ratio. The following settings were used to detect the fragment masses between 100 Da and 2250 Da within one second: 2.5 kV capillary, 30 V sample cone, 3 V extraction cone, 100 °C source temperature, 350 °C desolvation temperature, 50 L/h cone gas and 750 L/h desolvation gas in the MSe-, positive-, V-mode.

The resulting retention time in combination with the obtained mass was used to identify the distinct glycan structures, when the intensity was over the detection limit of 0.2%. Almost all peaks were assigned to common glycan structures according to their mass. Incorporation of fucose analogues was detected through a mass shift in the MS spectra by +9.99 Da for the 5-alkynylfucose derivative and +1.99 Da for the 2-fluorofucose derivative. The highest detected mass error within all experiments was ±3.53 ppm. Some undefined structures remained due to an inconclusive mass. For quantification of the glycan content, the relative peak area was determined using a fluorescence detector. Thus, the percentage of each glycoform was quantified by calculating the ratio of the peak area of each glycan to the sum of all peak areas. 

### 2.4. Statistical Analysis

Statistical analysis was performed using GraphPad Prism 6 (GraphPad Software Inc., San Diego, CA, USA). More precisely, the non-parametric Kruskal-Wallis test for multiple-group comparison with subsequent Dunn’s tests was performed. The comparison of two groups was realized by the Mann-Whitney test. *p*-values smaller than 0.05 were considered as significant.

## 3. Results

The reduction of core fucosylation in the Fc-part of antibodies leads to enhanced ADCC and is therefore the aim of several investigations in the literature. Supplementation of small molecules as additive to cell culture media is one possibility to achieve a reduction in fucosylation without further cell line engineering.

### 3.1. Cell Performance

Fed-batch cell culture experiments on a CHO clone producing an IgG1 were performed to assess the efficacy of the 2F- and 5-Alk fucose analogues on reducing fucosylation of the recombinant IgG1. For manufacture of therapeutic mAbs, it is critical to ensure that the inhibitors do not negatively impact cellular performance, such as lowering the viable cell density (VCD), the viability of the cultured cells or the volumetric titer (amount of IgG produced). Several metabolites and other parameters like LDH were monitored in the supernatant of the cell culture to study the physiological state of the cells. A control condition containing DMSO only was incorporated into the study to distinguish between the effect of DMSO and the impact of the fucose analogues, as the fucose analogues were dissolved in DMSO for addition to cell culture.

**Figure 2:**
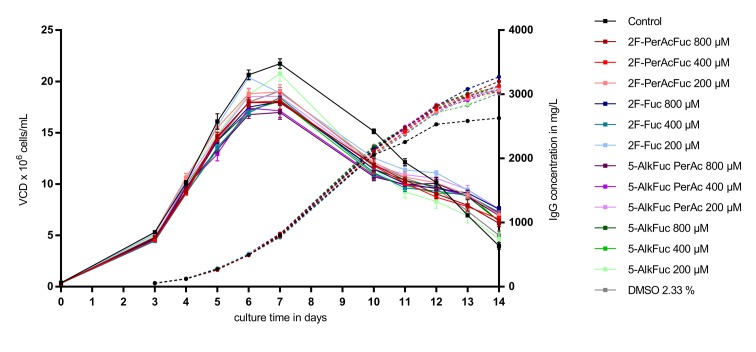
Viable cell density (VCD) and titer of Chinese hamster ovary (CHO) cells treated with fucose analogues. Four biological replicates were performed for each condition. The VCD is visualized as squares with a solid line and similar colors were used for the titer indicated with circles and a dashed line. Data are expressed as mean values ± standard error of the mean (SEM).

As visualized in Figure 2, supplementation of a feed containing 2.33% DMSO led to a reduction of the VCD (calculated as area under the curve) of about 11% compared to the control condition (*n* = 8). Independently of the applied concentration of 2F-Fuc and 5-AlkFuc, all conditions had a similar VCD compared to the DMSO-treated condition. The reduction was in a range between 5% and 15% and was suggested to be within the biological variability of fed-batch bioprocesses. Quantification of the IgG production showed a similar positive effect in all conditions containing 2.33% DMSO. The titer was about 6% to 10% higher and resulted in an increased specific productivity of about 25 ± 1% in all conditions containing DMSO. As VCD, IgG and the resulting specific productivity of fucose analogues were in a similar range than the DMSO control, they were thought to have no impact on cell parameters chosen in this study.

### 3.2. Glycosylation Profile

The glycosylation profile has an impact on efficacy and safety of therapeutic antibodies and is one of the main critical quality attributes. The impact of fucose analogues on the glycosylation profile of two biological replicates was investigated on day 7, 10 and 12.

**Figure 3:**
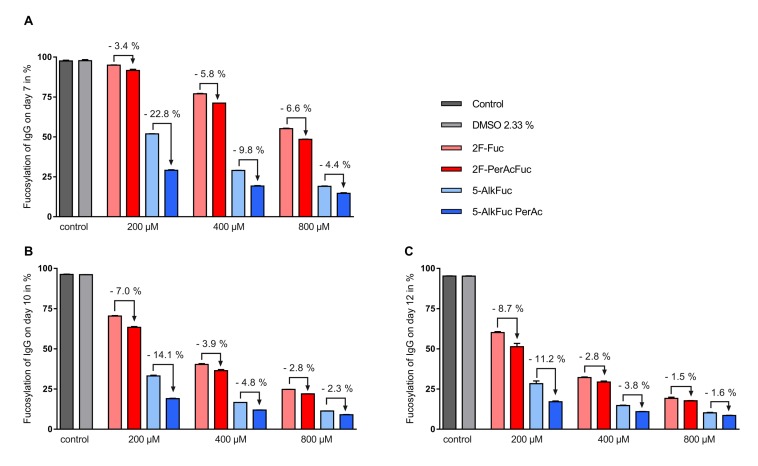
IgG fucosylation using two fucose analogues and the respective acetylated compounds. Feed was adjusted to 2.33% Dimethyl sulfoxide (DMSO) (except for the control) and added on day 3 (3%; *v*/*v*), day 5, 7 and 10 (6%; *v*/*v*). Fucosylation analysis on (**A**) day 7, (**B**) day 10 and (**C**) day 12 was obtained by UPLC-MS. Data are expressed as mean values ± SEM of two biological replicates. Statistical analysis was performed with all tested concentrations with and without peracetylation including data of additional experiments using various concentrations (data not shown). The data indicates a significant reduction of the IgG fucosylation compared to the control with a *p*-value < 0.0001 (*n* = 19) for 5-AlkFuc analogues and a *p*-value of 0.0050 (*n* = 19) for 2F-Fuc analogues.

The Fc-glycosylation profile of the IgG produced in untreated CHO cells (control) on day 12 consisted of 80.1% terminal *N*-acetylglucosamine (GlcNAc), 16.8% galactosylated structures and 3.0% high mannose glycans. 95.6% of the glycans were core fucosylated whereas terminal sialylation was mostly under the detection limit and is therefore considered as negligible (Figure 3). The main fucosylated forms were biantennary species with no galactose (G0F) with 77.0% as visualized in Table 1. The second most prevalent glycan structure was G1F with 15.5% relative abundance. Galactosylation of the second branch led to the di-galactosylated glycan G2F, which was detected in 0.9% of the glycans. Other fucosylated structures like G0F-N (1.8%) and G1F-N (0.4%) were less frequent. The smaller amount of afucosylated structures in the control condition were composed of Man5 (3.0%), G0 (0.8%) and G0-N (0.5%), while the galactosylated glycan structures G1 and G2 were below the detection limit. 

To assess the impact of fucose analogues on the glycosylation profile, the impact of the solvent used to dissolve the compounds needed to be excluded first. The applied DMSO concentration was 2.33% and showed no detectable changes of the glycosylation pattern compared to the control (Table 1). Total fucosylation was examined on days 7, 10 and 12, and all inhibitors tested were able to decrease IgG core fucosylation significantly. In general, a higher reduction of the fucosylation was achieved with both 5-Alk fucose analogues compared to the 2F-Fuc analogues. The most important decrease in core fucosylation was observed for the 800 µM peracetylated 5-AlkFuc condition with a final IgG core fucosylation of 8.6% (Figure 3, day 12). Comparison of acetylated versus the free-hydroxy fucose analogues showed that acetylation led to a larger decrease in core fucosylation, for both inhibitor types, at all concentrations tested (Figure 3). Interestingly, the effect of the acetylation was generally less important for increasing concentrations of the inhibitors. For example, acetylation for the 5-AlkFuc inhibitor (on day 12) led to 11.2%, 3.8% and 1.6% lower total fucosylated species at 200, 400 and 800 µM respectively, showing that acetylation is not necessary for higher amounts of derivatives. The only exception to this pattern was for the 2F-Fuc analogues measured on day 7, in which the opposite trend was observed. As the concentration of 2F-Fuc increased, acetylation lead to more pronounced reduction in core fucosylation (3.4% at 200 µM, 5.8% at 400 µM and 6.6% at 800 µM). 

Altogether, these results indicate that all fucose analogues can reduce total fucosylation, peracetylation improves the impact of the fucose analogues and increasing inhibitor concentrations reduces the impact of peracetylation. 

### 3.3. Incorporation of Fucose Analogues

**Figure 4:**
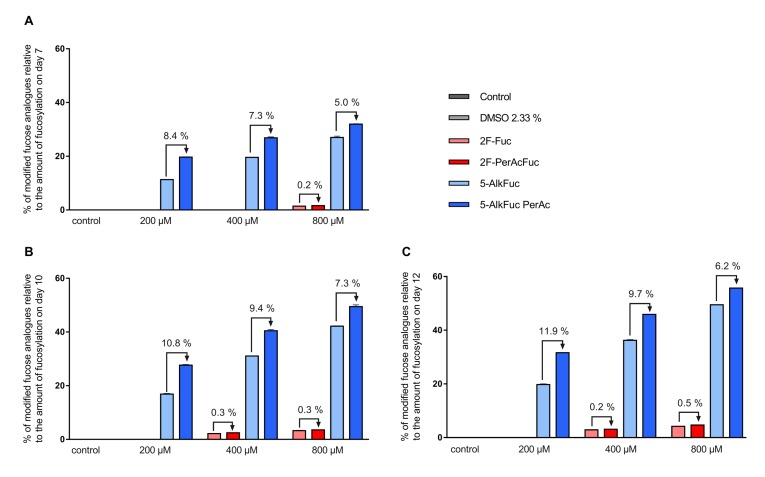
Relative incorporation of fucose analogues. Feed supplemented with fucose analogues was adjusted to 2.33% DMSO (except for the control) and added on day 3 (3%; *v*/*v*) day 5, 7 and 10 (6%; *v*/*v*). Glycosylation analysis on (**A**) day 7, (**B**) day 10 and (**C**) day 12 was obtained by UPLC-MS and incorporation was detected by a distinct mass-shift. The incorporation of the fucose analogues was calculated as sum of all glycan structures with incorporated fucose analogues relative to the amount of the fucosylation. Data are expressed as mean values ± SEM of two biological replicates.

Mass spectrometry data revealed that both fucose analogues are also incorporated into the antibody (this is henceforth indicated with an * after the respective glycan structure abbreviation). For simplicity, all fucosylation values provided so far have also included any incorporated fucose analogues. The fluorinated fucose was detected in the released glycans by a mass shift of +1.99 Da, while the alkylated fucose was recognized by a +9.99 Da shift. The detected mass shift of the fucose analogues is visualized in the supplementary data in Figure S1 and Figure S2 exemplarily for the G0F species. 5-AlkFuc led to higher incorporation of the fucose analogue compared to 2F-Fuc. 5% of the glycans showed 5-AlkFuc incorporation and 0.8% of the glycans showed 2F-Fuc incorporation, independently of the applied concentration. Due to higher total fucosylation for lower derivative concentrations, the relative values for incorporation varied, while absolute values were consistent. To visualize the concentration dependency, the relative incorporation of fucose analogues to the respective total fucosylation was calculated (Figure 4). For example, only 10% of the glycans remained core-fucosylated in the 800 µM 5-AlkFuc condition on day 12 (Figure 3), and 50% of these forms were chemically modified (Figure 4). The lower 5-AlkFuc concentrations 400 µM and 200 µM led to 37% and 20% relative incorporation, respectively. These results indicate a linear correlation between incorporation and the applied inhibitor concentration. Thus, higher 5-AlkFuc concentrations may lead to more undesired incorporation. 

No incorporation of 2F-Fuc was detected in the 200 µM treated condition on all days, when using a threshold of 0.2% of the peak area in the glycan analysis setting. The UPLC-MS data revealed low incorporation when reducing this threshold (data not shown). The highest incorporation of fluorinated fucose relative to the total fucosylation was detected on day 12 with 4.7%, when 800 µM 2F-PerAcFuc was applied. Altogether, a more effective reduction of the IgG fucosylation by 5-AlkFuc compared to 2F-Fuc was associated with a significant higher incorporation of the alkynylated fucose analogue. Therefore, higher 2F-Fuc concentrations may be more favorable compared to 5-AlkFuc.

The 5-AlkFuc analogue was detected in three different glycan structures, which have been identified as biantennary complex containing either zero or one galactose as listed in Table 2. The main detected specie was G0F* with a relative abundance of 4.6% on day 12. The second glycan that showed incorporation was G1F* with 0.8% and lastly G0F*-N. This trend indicates that incorporation of the analogues is not influenced by a preference of the FUT8 for an acceptor glycan. 

Incorporation of monosaccharide analogues may be desired e.g., in the context of antibody drug conjugation to define a specific conjugation position. In case of biosimilar development, the incorporation of analogues is rather undesired. These data highlight the importance of a proper glycan analytic enabling the detection of undesired incorporation. Additionally, the selection of an appropriate inhibitor as well as the applied concentration have to be selected carefully to achieve the intended final amount of core fucosylation.

## 4. Discussion

Acetylation of monosaccharides was reported to improve cell permeability by increasing hydrophobicity [17,18]. Therefore, modulation of the glycosylation pattern is often performed with acetylated derivatives of monosaccharides [19,20]. For instance, acetylated fucose analogues were reported as highly potent compounds to reduce IgG core fucosylation [10]. The data of Okeley et al. indicate that fucose analogues without acetylation might be applicable but were not investigated further [21]. Therefore, the impact of acetylation on the efficacy of the fucose analogues to reduce core fucosylation was analyzed in this work by comparing 2-fluorofucose and 5-alkynylfucose with and without acetylation. Increasing inhibitor concentrations and consecutive timepoints showed a reduced effect of acetylation.

The effect of acetylation on cellular entry has never been studied for fucose derivatives but was studied in the literature for *N*-acetylmannosamine (ManNAc) and several ManNAc analogues, commonly used as precursors for sialic acid and thereby sialylation. Results show that more ManNAc is present intracellularly when a fully acetylated form is added to the cell culture media, leading to up to 900-fold improved sialic acid production in cells. This study shows furthermore that the ManNAc concentration correlates linearly with sialic acid production for concentrations up to 25 mM whereas a non-linear relationship is described for fully acetylated ManNAc above 100 µM [22]. In contrast to the linear correlation observed for ManNAc and sialic acid, our results indicate a non-linear correlation between fucosylation and both the peracetylated and non-acetylated fucose analogues. The non-linear correlation may indicate either the usage of excessive fucose-analogue concentrations or non-linear uptake rates of the respective derivatives. This effect might be studied in detail by monitoring the intracellular concentrations of fucose analogues in combination with resulting GDP-fucose analogues but was beyond the scope of this study. 

The general low impact of acetylation detected in this study may be explained by differences in the mechanism of cellular transport when comparing fucose, mannosamine derivatives and other carbohydrates. In the literature, no ManNAc transporter has been described so far and such a transporter might not be essential for cells since ManNAc is endogenously produced using glucose as precursor [23]. Therefore, supplemented ManNAc is suggested to enter cells via passive diffusion. Similarly, peracetylation of 1-deoxy-N-pentynyl glucosamine was reported to improve the uptake of the glucosamine derivative due to enhanced passive diffusion [24]. In contrast, fucose as well as the analogues may be transported in cells via a transporter. The sodium/myo-inositol transporter slc5a3 is expressed in CHO cells and was reported to transport L-Fucose due to the high structural similarity to myo-inositol [25,26,27]. The hypothesis that fucose derivatives can be recognized by the transporter is further supported by the fact that fucose analogues are also recognized by other key enzymes in the fucosylation pathway such as fucokinases and GDP-L-fucose pyrophosphorylases. Both enzymes are converting the analogues into actual GDP-fucose-analogue inhibitors. Moreover, GDP-2F-Fuc was described as an inhibitor of GDP-mannose 4,6-dehydratase (GMD), and GDP-5-AlkFuc as an inhibitor of FX, both required for the de novo synthesis of fucose [10,15].

In this study, the reduction of fucosylation was more efficient using 5-AlkFuc, a known inhibitor of FX compared to 2F-Fuc, which is known to inhibit GMD. The high efficiency of 5-Alk-Fuc may result from the competitive FX inhibition by GDP-5-AlkFuc with a K_i_ of 2.9 µM [15]. In comparison, Kizuka et al. reported a rather low FUT8 inhibition by the activated sugar with a K_i_ of 91 µM but a K_M_ of 691 µM, allowing the incorporation of 5-AlkFuc into the glycan structure [15]. This higher incorporation in the glycan structure indicates that 5-AlkFuc is more likely to be tolerated by enzymes involved in the salvage pathway leading to higher intracellular GDP-5-AlkFuc concentrations. A higher intracellular GDP-5-AlkFuc pool is likely to result in enhanced feedback inhibition, compared to the feedback inhibition generated by 2F-Fuc, leading to a faster reduction of the native substrate pool and thereby reduced fucosylation. This trend of a lower GDP-Fuc pool was also suggested by the results of Okeley et al. [14]. Finally, the observed incorporation of 5-Alk-Fuc in the glycan structure confirms the tolerance of both, GDP-fucose transporter and FUT8 for the GDP-fucose analogue. In addition to a likely competitive FUT8 inhibition, the activated sugar can thus also be utilized as slow substrate.

According to literature data, differences in the frequency of incorporation may be due to the relative position of the modification, the nature of the modification, or more likely the interplay of both. Modifications of fucose at carbon positions C-2, C-5 and C-6 have been frequently reported in the literature [14,15,21,28], while modifications at other carbon positions are likely undesirable, as these may be required for enzyme binding [28]. By comparing the incorporation data of 6F-fucose obtained by Okeley et al., to our data on 2F-fucose, we can gain an insight into the binding modality of some enzymes in the fucosylation pathway. Our results showed a 0.8% incorporation of 2F-fucose whereas 6F-fucose resulted in >90% incorporation [21]. This suggest that the C-6 hydroxy is less critical in substrate recognition for the enzymes in this pathway than the C-2 hydroxy group. The importance of the hydroxyl group at carbon position C-2 for enzyme recognition was also suggested by McKenzie et al. [29]. 

Regarding the nature of the chemical modification, the 5-ethyl analogue led to more than 90% incorporation, whereby 5-AlkFuc showed only 5% incorporation [21]. Moving the alkynyl moiety further from the carbohydrate ring via insertion of a methylene group (producing 6-AlkFuc) led to a considerably higher incorporation of the fucose analogue into the antibody, which allows the application as biomarker [30]. Another factor likely to impact the extent of incorporation of the derivative into the glycan structure are the changes in electronegativity imparted by the modification. Replacing a hydrogen on the C-6 of fucose with chlorine or sulphur led to 80% and 70% incorporation of these analogues, respectively [14]. Along with the electronegativity, altered size of the respective residue may have an additional impact. Allen et al. reported decreasing incorporation of all glycans from 70% with 6-fluorofucose to about 11% using 6,6-difluorofucose and about 1% when 6,6,6-trifluorofucose was applied. The group suggested a destabilization of the oxocarbenium ion-like transition state within the FUT8 enzyme when using the trifluorofucose as reason for the low incorporation [31]. Altogether this data suggests that bulkier modifications on position 6 might reduce incorporation. An efficient reduction of core-fucosylation using 6,6,6-trifluorofucose was also reported by McKenzie et al. using a murine hybridoma cell line instead of the commonly used CHO cell line [29]. Still, different cell lines may respond differently to the derivatives due to slight changes in enzyme structure, changed affinity toward substrates or as a result of different amounts and structure of fucosyltransferases. No incorporation of 2F-Fuc was reported on the cell surface of HL-60 cells, whereby low incorporation was detected when using transfected CHO Lec13 cells, similar to our results using CHOK1 cells. Therefore, it was suggested that a slightly changed FUT8 activity or selectivity between cell lines may lead to different amounts of incorporation [10]. Additionally, Lec13 cells are lacking the de novo GDP-fucose synthesis, thus the ratio between native FUT8 substrate and GDP-fucose-analogue may have an impact on the incorporation. Investigation of the GDP-fucose ratio to the respective analogue may give further insight into this dependency. In addition, substrate ratio may depend on the occurence of other fucosyltransferase subtypes, since they use the same activated sugar nucleotide GDP-Fuc as substrate [32].

Ultimately, the extent of fucose analogue incorporation may depend on the cell line, substrate specificity and efficacy of the enzymes (especially FUT8) in the fucosylation pathway, different transport rates or different inhibition of either GMD or FX by changing the ratio of natural/modified substrate. Therefore, the incorporation of fucose analogues is complex and multifactorial and may not be easily predicted.

Overall, chemical modification of the fucose structure is a powerful method to reduce core fucosylation without cell engineering, but a careful analysis of potential analogue incorporation is required. Furthermore, an investigation of the effect of the fucose modification on the binding affinity to the respective Fc-receptor is important. These tests go beyond the scope of this work and will be part of future studies. As long as the impact of modified monosaccharides within antibodies is uncertain, incorporation of any non-native sugar has to be prevented, especially if the resulting antibodies are applied as therapeutic drugs. 

## Figures and Tables

**Table 1:** Glycan structures of 2-fluorofucose with and without acetylation (200, 400 and 800 µM) compared to both control conditions.

		Control			2.33% DMSO			200 µM 2F-Fuc			200 µM 2F-Fuc PerAc			400 µM 2F-Fuc			400 µM 2F-Fuc PerAc			800 µM 2F-Fuc			800 µM 2F-Fuc PerAc		
Name ‡	Structure †	D7 (*n* = 5)	D10 (*n* = 6)	D12 (*n* = 6)	D7 (*n* = 3)	D10 (*n* = 3)	D12 (*n* = 3)	D7 (*n* = 2)	D10 (*n* = 2)	D12 (*n* = 2)	D7 (*n* = 2)	D10 (*n* = 2)	D12 (*n* = 2)	D7 (*n* = 2)	D10 (*n* = 2)	D12 (*n* = 2)	D7 (*n* = 2)	D10 (*n* = 2)	D12 (*n* = 2)	D7 (*n* = 2)	D10 (*n* = 2)	D12 (*n* = 2)	D7 (*n* = 4)	D10 (*n* = 2)	D12 (*n* = 5)
G0F	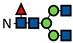	80.4 ± 2.1	77.5 ± 1.0	77.0 ± 0.9	81.8 ± 1.6	78.8 ± 1.1	77.1 ± 0.1	80.9 ± 0.7	57.7 ± 0.2	49.0 ± 0.2	78.5 ± 0.4	52.1 ± 0.3	42.0 ± 1.9	65.7 ± 0.1	33.1± 0.04	26.3 ± 0.1	59.2 ± 1.1	29.7 ± 0.3	23.7 ± 0.5	46.4 ± 0.1	20.5 ± 0.5	15.6 ± 0.7	38.6 ± 2.8	17.9 ± 0.5	13.1 ± 1.5
G0F *	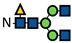	-	-	-	-	-	-	-	-	-	-	-	-	-	0.9 ± 0.03	0.9 ± 0.03	-	0.9 ± 0.05	0.9 ± 0.01	0.8 ± 0.01	0.8 ± 0.06	0.8 ± 0.01	0.8 ± 0.02	0.8 ± 0.03	0.8 ± 0.1
G1F	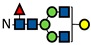	15.6 ± 2.5	16.4 ± 1.4	15.5 ± 1.2	14.2 ± 2.0	14.9 ± 1.3	14.9 ± 0.8	12.3 ± 0.5	10.5 ± 0.1	8.7 ± 0.6	11.5 ± 0.8	9.3 ± 0.1	7.4 ± 0.8	9.9 ± 0.4	5.7 ± 0.2	4.4 ± 0.4	9.2 ± 0.6	5.3 ± 0.2	4.3 ± 0.4	7.4 ± 0.1	3.2 ± 0.7	2.8 ± 0.3	7.4 ± 1.0	3.0 ± 0.4	2.7 ± 0.8
G1F *	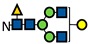	-	-	-	-	-	-	-	-	-	-	-	-	-	-	-	-	-	-	-	-	-	-	-	-
G0F-N	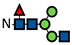	0.9 ± 0.1	1.4 ± 0.3	1.8 ± 0.2	0.8 ± 0.2	1.5 ± 0.4	2.1 ± 0.2	0.7 ± 0.02	1.2 ± 0.01	1.3 ± 0.01	0.7 ± 0.2	1.1 ± 0.1	1.2 ± 0.01	0.8 ± 0.1	-	-	0.8	-	-	-	-	-	-	-	-
G0F *-N	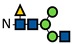	-	-	-	-	-	-	-	-	-	-	-	-	-	-	-	-	-	-	-	-	-	-	-	-
G1F-N	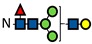	-	0.3 ± 0.1	0.4 ± 0.04	-	0.3 ± 0.1	0.4 ± 0.1	-	-	-	-	-	-	-	-	-	-	-	-	-	-	-	-	-	-
G2F	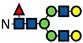	0.8 ± 0.2	0.9 ± 0.1	0.9 ± 0.1	0.7 ± 0.1	0.8 ± 0.2	0.9 ± 0.03	0.6 ± 0.1	0.7 ± 0.01	0.6 ± 0.01	0.5 ± 0.1	0.6 ± 0.1	0.4 ± 0.02	0.5 ± 0.03	0.4 ± 0.02	0.3 ± 0.02	0.5 ± 0.01	0.4 ± 0.01	0.3 ± 0.01	0.4 ± 0.04	0.3 ± 0.02	-	0.4 ± 0.1	0.3 ± 0.02	-
G0	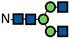	0.7 ± 0.1	0.8 ± 0.1	0.8 ± 0.1	0.8 ± 0.04	0.7 ± 0.1	0.8 ± 0.1	3.8 ± 0.1	23.4 ± 0.5	30.8 ± 0.6	6.0 ± 0.05	29.1 ± 0.1	36.3 ± 0.7	20.2 ± 0.5	48.7 ± 0.6	54.6 ± 0.7	25.5 ± 0.6	52.1 ± 1.4	56.9 ± 1.2	39.4 ± 0.03	62.1 ± 0.4	64.0 ± 1.3	46.0 ± 1.0	64.0 ± 0.8	68.0 ± 2.5
G1	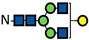	-	-	-	-	-	-	0.3 ± 0.01	2.6 ± 0.03	3.4 ± 0.1	0.4 ± 0.02	3.3 ± 0.1	3.9 ± 0.4	1.6 ± 0.02	5.6 ± 0.04	6.2 ± 0.3	2.2 ± 0.1	6.1 ± 0.04	6.6 ± 0.3	3.6 ± 0.04	6.9 ± 0.1	7.4 ± 0.5	5.0 ± 0.9	7.4 ± 0.08	8.8 ± 1.1
G2	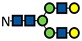	-	-	-	-	-	-	-	-	-	-	-	-	-	-	-	-	-	0.21	-	0.2 ± 0.01	0.2 ± 0.03	-	0.2 ± 0.01	0.2 ± 0.1
Man5	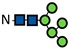	0.7 ± 0.2	1.8 ± 0.4	3.0 ± 0.3	0.7 ± 0.1	2.1 ± 0.4	3.2 ± 0.3	0.8 ± 0.1	2.3 ± 0.1	3.4 ± 0.1	0.8 ± 0.01	2.3 ± 0.1	3.4 ± 0.3	0.8 ± 0.05	2.4 ± 0.1	3.6 ± 0.2	0.9 ± 0.01	2.4 ± 0.05	3.6 ± 0.2	0.9 ± 0.02	2.5 ± 0.01	3.4 ± 0.2	0.9 ± 0.04	2.5 ± 0.04	3.5 ± 0.2
G0-N	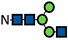	0.2 ± 0.03	0.2 ± 0.2	0.5 ± 0.1	0.3	0.3 ± 0.3	0.4 ± 0.1	0.3	1.2 ± 0.05	2.1 ± 0.3	0.3 ± 0.2	1.4 ± 0.2	3.4 ± 1.7	0.4 ± 0.2	2.0 ± 0.1	2.9 ± 0.3	0.5 ± 0.03	2.1 ± 0.01	2.9 ± 0.4	0.8 ± 0.1	2.4 ± 0.04	3.7 ± 1.0	0.8 ± 0.1	2.5 ± 0.1	2.8 ± 0.5

† 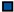 represents *N*-acetylglucosamine (GlcNAc); 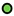 represents mannose; 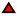 represents fucose; 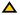 represents fucose analogues; 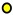 represents galactose. ‡ respective fucose-analogue is indicated with *. The Glycan species were identified through the mass. The percentages of various glycans were calculated according to the relative area of each glycan species as detected using RapiFluor™ (Waters) as labelling reagent. Data are presented as mean values ± SEM and without SEM means that the peaks were only detected in one replicate.

**Table 2:** Glycan structures of 5-alkynylfucose with and without acetylation (200, 400 and 800 µM) compared to both control conditions.

		Control			2.33% DMSO			200 µM 5-AlkFuc			200 µM 5-AlkFuc PerAc			400 µM 5-AlkFuc			400 µM 5-AlkFuc PerAc			800 µM 5-AlkFuc			800 µM 5-AlkFuc PerAc		
Name ‡	Structure †	D7 (*n* = 5)	D10 (*n* = 6)	D12 (*n* = 6)	D7 (*n* = 3)	D10 (*n* = 3)	D12 (*n* = 3)	D7 (*n* = 2)	D10 (*n* = 2)	D12 (*n* = 2)	D7 (*n* = 2)	D10 (*n* = 2)	D12 (*n* = 2)	D7 (*n* = 2)	D10 (*n* = 2)	D12 (*n* = 2)	D7 (*n* = 2)	D10 (*n* = 2)	D12 (*n* = 2)	D7 (*n* = 2)	D10 (*n* = 2)	D12 (*n* = 2)	D7 (*n* =2)	D10 (*n* = 2)	D12 (*n* =2)
G0F	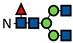	80.4 ± 2.1	77.5 ± 1.0	77.0 ± 0.9	81.8 ± 1.6	78.8 ± 1.1	77.1 ± 0.1	37.9 ± 0.02	21.8 ± 0.5	17.8 ± 1.3	19.4 ± 0.2	11.2 ± 0.2	9.4 ± 0.3	19.4 ± 0.1	9.4 ± 0.2	7.6 ± 0.1	11.4 ± 0.1	5.8 ± 0.1	4.8 ± 0.1	11.5 ± 0.01	5.2 ± 0.2	4.1 ± 0.1	8.0 ± 0.1	3.7 ± 0.1	3.0 ± 0.01
G0F *	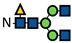	-	-	-	-	-	-	4.9 ± 0.1	4.7 ± 0.01	4.7 ± 0.2	5.0 ± 0.01	4.5 ± 0.02	4.6 ± 0.1	5.0 ± 0.03	4.4 ± 0.01	4.6 ± 0.02	4.5 ± 0.02	4.1 ± 0.05	4.3 ± 0.01	4.5 ± 0.01	4.0 ± 0.03	4.3 ± 0.04	4.1 ± 0.03	3.8 ± 0.03	4.1 ± 0.04
G1F	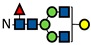	15.6 ± 2.5	16.4 ± 1.4	15.5 ± 1.2	14.2 ± 2.0	14.9 ± 1.3	14.9 ± 0.8	7.2 ± 0.2	4.9 ± 0.1	4.1 ± 0.7	3.5 ± 0.6	2.3 ± 0.1	2.0 ± 0.2	3.5 ± 0.4	2.0 ± 0.01	1.6 ± 0.2	2.7 ± 0.1	1.3 ± 0.01	1.1 ± 0.03	2.4 ± 0.34	1.2 ± 0.02	1.0 ± 0.1	2.0 ± 0.3	0.9 ± 0.03	0.8 ± 0.1
G1F *	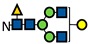	-	-	-	-	-	-	0.9 ± 0.04	0.9 ± 0.01	0.9 ± 0.1	0.7 ± 0.04	0.7 ± 0.01	0.8 ± 0.1	0.7 ± 0.02	0.7 ± 0.01	0.7 ± 0.04	0.7 ± 0.2	0.7 ± 0.01	0.7 ± 0.1	0.6 ± 0.04	0.8 ± 0.04	0.8 ± 0.2	0.6 ± 0.2	0.7 ± 0.03	0.7 ± 0.02
G0F-N	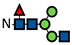	0.9 ± 0.1	1.4 ± 0.3	1.8 ± 0.2	0.8 ± 0.2	1.5 ± 0.4	2.1 ± 0.2	0.7 ± 0.1	0.6 ± 0.03	0.7 ± 0.02	0.3 ± 0.02	0.3 ± 0.01	0.3	0.3 ± 0.1	-	0.2 ± 0.01	-	-	-	-	-	-	-	-	-
G0F *-N	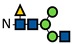	-	-	-	-	-	-	-	-	0.3 ± 0.2	-	-	0.2	-	-	0.2 ± 0.01	-	-	-	-	-	-	-	-	-
G1F-N	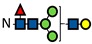	-	0.3 ± 0.1	0.4 ± 0.04	-	0.3 ± 0.1	0.4 ± 0.1	-	-	-	-	-	-	-	-	-	-	-	-	-	-	-	-	-	-
G2F	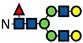	0.8 ± 0.2	0.9 ± 0.1	0.9 ± 0.1	0.7 ± 0.1	0.8 ± 0.2	0.9 ± 0.03	0.3 ± 0.02	0.3 ± 0.02	0.2 ± 0.03	0.2	-	-	0.2	-	-	-	-	-	-	0.29	-	-	-	-
G0	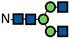	0.7 ± 0.1	0.8 ± 0.1	0.8 ± 0.1	0.8 ± 0.04	0.7 ± 0.1	0.8 ± 0.1	41.8 ± 0.4	53.7 ± 0.3	54.2 ± 2.2	62.8 ± 0.9	66.6 ± 0.2	66.9 ± 0.2	63.1 ± 0.6	69.2 ± 0.3	70.0 ± 0.6	70.6 ± 1.0	73.8 ± 0.2	73.7 ± 0.2	71.7 ± 0.7	73.6 ± 0.6	74.1 ± 0.9	74.7 ± 0.5	75.9 ± 0.7	75.6 ± 0.4
G1	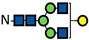	-	-	-	-	-	-	4.3 ± 0.1	6.7 ± 0.1	6.5 ± 0.7	6.1 ± 0.1	7.7 ± 0.1	7.6 ± 0.6	5.9 ± 0.03	8.2 ± 0.03	7.9 ± 0.6	6.9 ± 0.1	8.4 ± 0.1	8.1 ± 0.3	7.0 ± 0.1	8.7 ± 0.2	8.6 ± 0.5	7.2 ± 0.4	8.7 ± 0.3	8.4 ± 0.5
G2	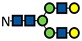	-	-	-	-	-	-	-	-	0.2	0.2	0.3 ± 0.01	0.3 ± 0.03	-	0.3 ± 0.01	0.3 ± 0.1	0.2	0.3 ± 0.01	0.3 ± 0.01	-	0.3 ± 0.02	0.3 ± 0.1	0.2 ± 0.01	0.3 ± 0.01	0.3 ± 0.03
Man5	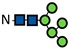	0.7 ± 0.2	1.8 ± 0.4	3.0 ± 0.3	0.7 ± 0.1	2.1 ± 0.4	3.2 ± 0.3	0.8 ± 0.04	2.2 ± 0.1	3.1 ± 0.4	0.9 ± 0.04	2.3 ± 0.1	3.4 ± 0.3	0.8 ± 0.01	2.3 ± 0.01	3.4 ± 0.2	1.0 ± 0.03	2.4 ± 0.01	3.4 ± 0.2	0.9 ± 0.01	2.5 ± 0.04	3.5 ± 0.2	1.0 ± 0.03	2.5 ± 0.02	3.6 ± 0.2
G0-N	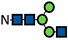	0.2 ± 0.03	0.2 ± 0.2	0.5 ± 0.1	0.3	0.3 ± 0.3	0.4 ± 0.1	0.9 ± 0.1	2.2 ± 0.1	3.5 ± 1.5	1.0 ± 0.01	2.4 ± 0.1	1.0 ± 0.01	1.0 ± 0.1	2.4 ± 0.04	3.2 ± 0.4	1.0 ± 0.1	2.5 ± 0.02	3.2 ± 0.3	1.0 ± 0.02	2.4 ± 0.03	3.2 ± 0.4	3.3 ± 0.6	2.6 ± 0.1	3.3 ± 0.3

† 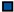 represents GlcNAc; 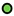 represents mannose; 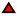 represents fucose; 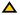 represents fucose analogues; 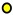 represents galactose. ‡ respective fucose-analogue is indicated with *. The Glycan species were identified through the mass. The percentages of various glycans were calculated according to the relative area of each glycan species as detected using RapiFluor™ as labelling reagent. Data are presented as mean values ± SEM and without SEM means that the peaks were only detected in one replicate.

## Supplementary Materials

The following are available online at https://www.mdpi.com/2073-4468/8/1/9/s1, Figure S1: MSe spectra overlay obtained at RT 14.50 min for G0F (black) and RT 13.89 min for G0F * with 5-alkynylfucose incorporated (blue) highlighting a 9.99 Da mass shift, Figure S2 MSe spectra overlay obtained at RT 14.50 min for G0F (black) and RT 12.50 min for G0F * with 2-fluorofucose incorporated (red) highlighting a 1.99 Da mass shift.

## Nomenclature

2-deoxy-2-fluorofucose	2F-Fuc
2F-Peracetyl-fucose	2F-PerAcFuc
5-alkynylfucose	5-AlkFuc
5-alkynylfucose peracetylated	5-AlkFuc PerAc
6-fluorofucose	6F-Fuc
antibody-dependent cellular cytotoxicity	ADCC
chinese hamster ovary cells	CHO cells
critical quality attribute	cQA
dimethyl sulfoxide	DMSO
Fucosyltransferase 8	FUT8
Guanosine diphosphate	GDP
GDP-4-keto-6-deoxymannose 3,5-epimerase-4-reductase	FX
GDP-mannose 4,6-dehydratase	GMD
monoclonal antibodies	mAbs
*N*-acetylglucosamine	GlcNAc
*N*-acetylmannosamine	ManNAc
standard error of the mean	SEM
ultra-performance liquid chromatography coupled to a mass spectrometer	UPLC-MS
viable cell density	VCD
