# Supplementary material for: Impact of Acetylated and Non-Acetylated Fucose Analogues on IgG Glycosylation
Source: Antibodies (Basel). 2019 Jan 10;8(1):9. doi: 10.3390/antib8010009 (PMC6640710; doi:10.3390/antib8010009)
Supplement: Supplementary file 1 [file antibodies-08-00009-s001.pdf]

Supplementary data:

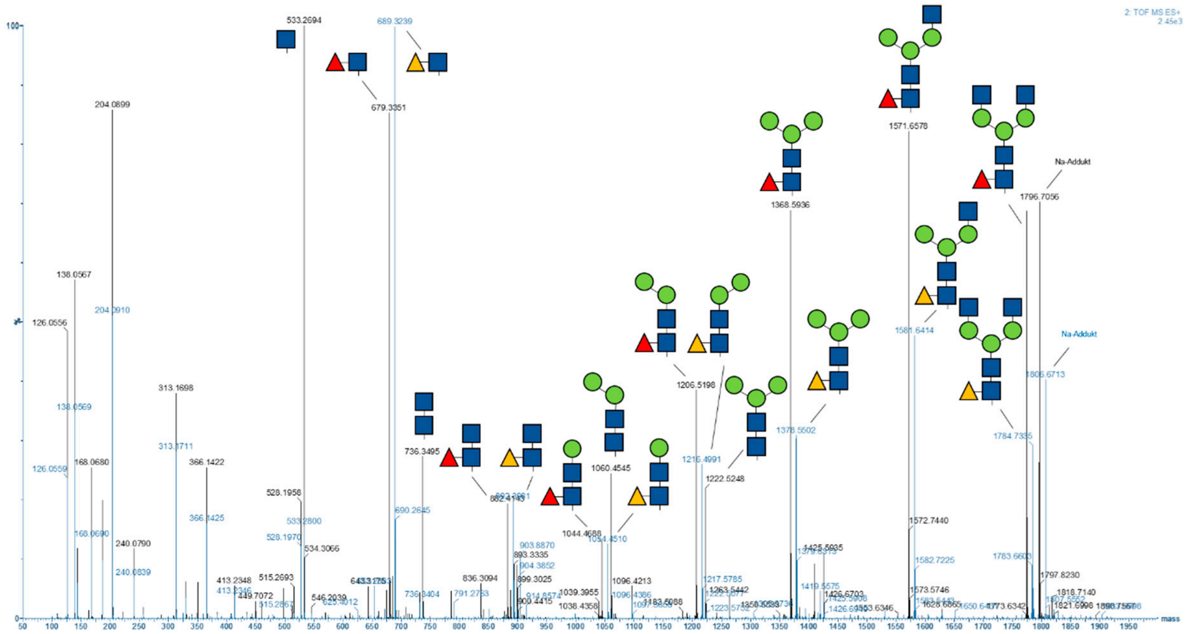

Figure S1. MSe spectra overlay obtained at RT 14.50 min for G0F (black) and RT 13.89 min for G0F * with 5-alkynylfucose incorporated (blue) highlighting a 9.99 Da mass shift. All molecular ions are $[M+H]^+$ containing the RapiFluor-MS™ tag from Waters.

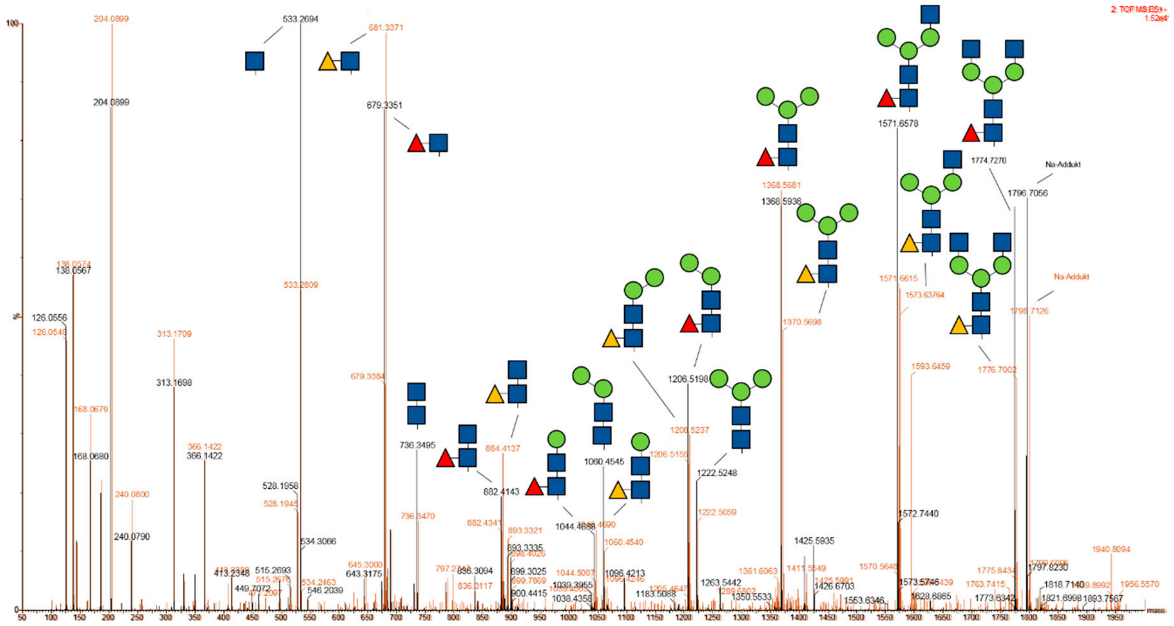

Figure S2. MSe spectra overlay obtained at RT 14.50 min for G0F (black) and RT 12.50 min for G0F * with 2-fluorofucose incorporated (red) highlighting a 1.99 Da mass shift. All molecular ions are $[M+H]^+$ containing the RapiFluor-MS™ tag from Waters. .
